# Investigation of the InAs/GaAs Quantum Dots’ Size: Dependence on the Strain Reducing Layer’s Position

**DOI:** 10.3390/ma8084699

**Published:** 2015-07-24

**Authors:** Manel Souaf, Mourad Baira, Olfa Nasr, Mohamed Helmi Hadj Alouane, Hassen Maaref, Larbi Sfaxi, Bouraoui Ilahi

**Affiliations:** 1 Laboratoire de Micro-Optoélectronique et Nanostructures, Faculté des Sciences, Avenue de l’environnement, Université de Monastir, Monastir 5019, Tunisia; E-Mails: manelsouaf@yahoo.fr (M.S.); mourad.baira@isimm.rnu.tn (M.B.); olfaa.nasr@gmail.com (O.N.); helmi.alouane@yahoo.fr (M.H.H.A); hassen.maaref@fsm.rnu.tn (H.M.); larbi.sfaxi@fsm.rnu.tn (L.S.); 2 King Saud University, Department of Physics & Astronomy, College of Sciences, P.O. 2455, Riyadh 11451, Saudi Arabia

**Keywords:** modeling, quantum dots, strain reducing layer

## Abstract

This work reports on theoretical and experimental investigation of the impact of InAs quantum dots (QDs) position with respect to InGaAs strain reducing layer (SRL). The investigated samples are grown by molecular beam epitaxy and characterized by photoluminescence spectroscopy (PL). The QDs optical transition energies have been calculated by solving the three dimensional Schrödinger equation using the finite element methods and taking into account the strain induced by the lattice mismatch. We have considered a lens shaped InAs QDs in a pure GaAs matrix and either with InGaAs strain reducing cap layer or underlying layer. The correlation between numerical calculation and PL measurements allowed us to track the mean buried QDs size evolution with respect to the surrounding matrix composition. The simulations reveal that the buried QDs’ realistic size is less than that experimentally driven from atomic force microscopy observation. Furthermore, the average size is found to be slightly increased for InGaAs capped QDs and dramatically decreased for QDs with InGaAs under layer.

## 1. Introduction

InAs self-assembled QDs formed by Stranski-Krastanov growth mode have been a subject of intense research activity towards the development of innovative devices such as QDs lasers [1,2], light emitters and detectors [3,4,5,6]. More recently, this kind of QDs has been found to be promising for photovoltaic applications [7]. However, the efficient exploitation of the self-assembled QDs towards high performance devices relay on the best optimization of their size dispersion and control of their emission energy. Accordingly, it is important to understand the impact of the capping and/or the underling layer matrix composition on the evolution of the QDs size and electronic structure. Several methods, based on the strain engineering, have been reported [8,9,10]. Indeed, replacing the GaAs surrounding material by InGaAs alloy leads to partial strain relief-induced modification of confinement potential allowing to manipulate inter and intraband transition energies in InAs QDs [11,12]. The SRL has been employed as a QDs capping layer [13], underling layer [14], or a combination between these two approaches [15]. Despite the numerous theoretical and experimental reports on the impact of the SRL on the optical and structural properties of the QDs [16,17,18,19,20,21,22,23], less attention has been devoted to the combined theoretical and optical spectroscopy investigation of the impact of the InAs QDs position with respect to the SRL on the evolution of the buried dots’ size. Actually, it is well established that the buried InAs QDs undergo a compositional change duo to In segregation and *in-situ* intermixing [20,24,25]. Accordingly, the accurate modeling of the QDs electronic properties requires the knowledge of the exact In compositional profile [23,26]. In the meanwhile, with the absence of such data, the combination of photoluminescence spectroscopy and numerical calculation of the InAs QDs’ electronic structure could constitute an alternative solution allowing a comprehensive investigation of the asymptotic buried dots’ size evolution depending on the surrounding materials composition.

In this context, we report on a numerical and experimental investigation of the effect of strain reducing layer position on the size and electronic properties of the InAs/GaAs QDs system. The theoretical estimation explains well the observed experimental results.

## 2. Results and Discussion

**Figure 1:**
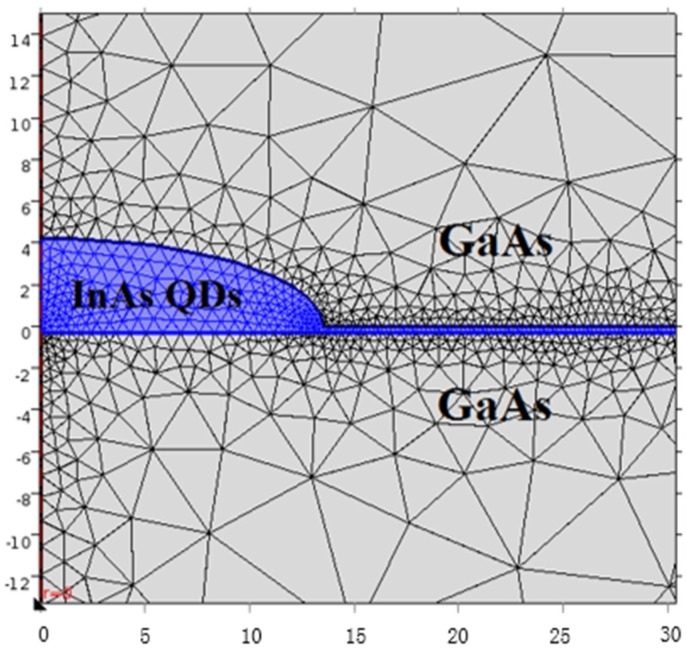
Schematic presentation of the zoomed lens shaped InAs quantum dots (QD) in a GaAs matrix. Also shown is the adaptive mesh refinement around the QD and the wetting layer.

Solving the Schrödinger equation with single band effective mass approximation by using the finite element methods (FEM) provided by COMSOL Multiphysics software, [27] affords a numerical estimation of the energy levels and corresponding wave functions. In this calculation, the InAs QDs are considered to be lens shaped as shown by the Figure 1.

The Schrödinger equation in cylindrical coordinates is given by:

(1) $$\frac{-\hbar^{2}}{2m^{*}}\left(\frac{\partial^{2}}{\partial r^{2}}+\frac{1}{r}\frac{\partial }{\partial r}+\frac{1}{r^{2}}\frac{\partial^{2}}{\partial \theta^{2}}+\frac{\partial^{2}}{\partial z^{2}}\right)\psi \left(r,\theta ,z\right)+V\psi \left(r,\theta ,z\right)=E\psi \left(r,\theta ,z\right)\;$$

where *E* represents the electron (hole) energy of state, m* electron (hole) effective masse, ħ the reduced Planck constant, θ is the azimuthal angle varied from 0 to 2π, *r* and *z* are, respectively, the radial and axial coordinates. Due to the rotational symmetric of the system, the Schrödinger equation can be reduced to a two dimensional problem were ψ(r,θ,z) can be written in the following form:

(2) ψ(r,ϕ,z)=χ(r,z)Ф(θ)

By dividing Equations (1) by (2) and then multiplying by *r^2^*, we obtain:

(3) $$\frac{-\hbar^{2}}{2m^{*}}\left(\frac{r^{2}}{\text{χ}\left(\text{r},\text{z}\right)}\frac{\partial^{2}\text{χ}\left(\text{r},\text{z}\right)}{\partial r^{2}}+\frac{r}{\text{χ}\left(\text{r},\text{z}\right)}\frac{\partial \text{χ}\left(\text{r},\text{z}\right)}{\partial r}++\frac{\partial^{2}\text{χ}\left(\text{r},\text{z}\right)}{\partial z^{2}}\right)+\left(V-E\right)r^{2}=\frac{\hbar^{2}}{2m^{*}}\frac{1}{Ф\left(\text{θ}\right)}\frac{\partial^{2}Ф\left(\text{θ}\right)}{\partial \theta^{2}}$$

the right part can be written as follows:

(4) $$\frac{\hbar^{2}}{2m^{*}}\frac{1}{Ф\left(\text{θ}\right)}\frac{\partial^{2}Ф\left(\text{θ}\right)}{\partial \theta^{2}}=\frac{-\hbar^{2}}{2m^{*}}n^{2}\;$$

where *n* is the orbital quantum number that must be integer:

(5) $$\frac{-\hbar^{2}}{2m^{*}}\left(\frac{r^{2}}{\text{χ}\left(\text{r},\text{z}\right)}\frac{\partial^{2}\text{χ}\left(\text{r},\text{z}\right)}{\partial r^{2}}+\frac{r}{\text{χ}\left(\text{r},\text{z}\right)}\frac{\partial \text{χ}\left(\text{r},\text{z}\right)}{\partial r}++\frac{\partial^{2}\text{χ}\left(\text{r},\text{z}\right)}{\partial z^{2}}\right)+\left(V-E\right)r^{2}+\frac{\hbar^{2}}{2m^{*}}n^{2}=0$$

when multiplying this equation by *χ(r,z)/r^2^*, we find the following equation:

(6) $$\frac{-\hbar^{2}}{2m^{*}}(\frac{\partial^{2}\text{χ}\left(\text{r},\text{z}\right)}{\partial r^{2}}+\frac{\partial^{2}\text{χ}\left(\text{r},\text{z}\right)}{\partial z^{2}})+(V+\frac{1}{r^{2}}\frac{\hbar^{2}}{2m^{*}}n^{2})\text{χ}\left(\text{r},\text{z}\right)-\frac{\hbar^{2}}{2m^{*}}\frac{1}{r}\frac{\partial \text{χ}\left(\text{r},\text{z}\right)}{\partial r}=E\text{χ}\left(\text{r},\text{z}\right)$$

To calculate the energy states and theirs wave functions for electron/hole in the QD, Dirichlet conditions are applied where the wave function must be zero (outside the considered domain) and the Neumann conditions are employed for the internal boundaries to ensure the continuity of the wave function. One of the major advantages of FEM is the flexibility to vary the desired precision over the entire domain by controlling the quality of the mesh. Accordingly, an adaptive mesh refinement has been employed as shown by the Figure 1.

The impact of strain on the carrier confinement due to the lattice mismatch between InAs and GaAs materials is also taken into account for the calculation [20]. Hydrostatic and uniaxial strains are given by the following expressions:

(7) $$\epsilon_{h}=\epsilon_{xx}+\epsilon_{yy}+\epsilon_{zz}$$

and

(8) $$\epsilon_{b}=\epsilon_{zz}-\frac{1}{2}\left(\epsilon_{xx}+\epsilon_{yy}\right)$$

where ε*_b_*, ε*_h_* are hydrostatics and uniaxial strain respectively. The lattice mismatch between InAs and GaAs is given by:

(9) $$\epsilon_{xx}=\epsilon_{yy}=\frac{a_{GaAs}-a_{InAs}}{a_{InAs}}$$

and

(10) $$\epsilon_{zz}=-2\frac{C_{12}}{C_{11}}\epsilon_{xx}$$

The strained effect on conduction band is $\delta E_{c}=a_{c}\epsilon_{h}$ and for valence band is

(11) $$\;\text{δ}E_{v}=a_{v}\epsilon_{h}-\frac{1}{2}b\epsilon_{b}$$

then the strained band gap energy (*Eg*) can be written as:

(12) $${\text{Eg}}_{\text{InAs}-\text{str}}={\text{Eg}}_{\text{InAs}-\text{unstr}}+\delta E_{c}-\text{δ}E_{v}\;$$

where *Eg_InAs-unstr_* is the unstrained InAs band gap energy and *Eg_InAs-str_* presents the strained one. The parameters used in this numerical approach are summarized in Table 1 [28].

**Table 1:** InAs and GaAs parameters used for the calculation.

Material	m_e_^*^ (m_0_)	m_h_^*^ (m_0_)	C_11_ 10^11^ (dyn cm^−2^)	C_12_ 10^11^ (dyn cm^−2^)	( *a*_c_−*a*_v_) (eV)	b (eV)
InAs	0.023	0.41	8.32	4.52	−4.08	−1.8
GaAs	0.068	0.5	12.21	5.66	−6	−2

The single particle optical transition energies E*_i_* can be deduced by the following equation:

(13) $$E_{i}=e_{i}+Eg_{InAs-Str}+h_{i}$$

where *e_i_* (*h_i_*) correspond to the electron (hole) confined state.

**Figure 2:**
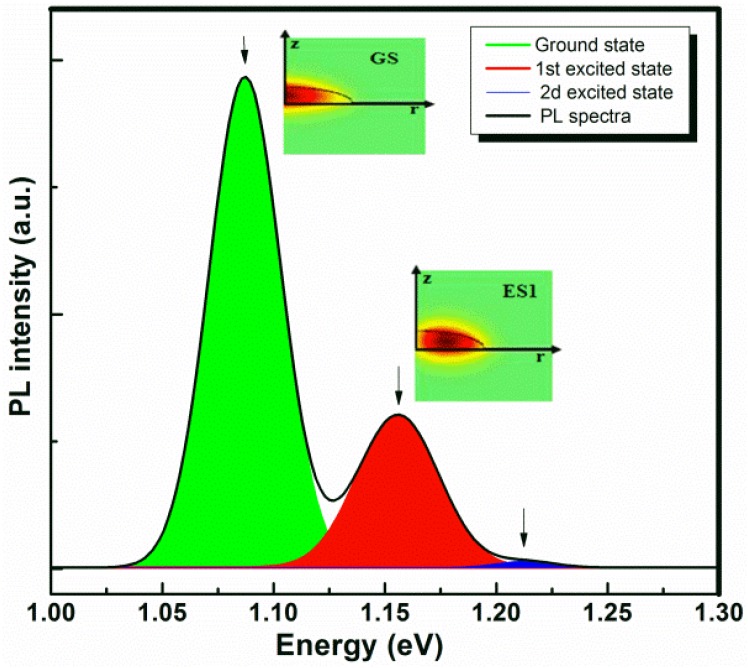
12 K photoluminescence spectroscopy (PL) spectra taken with an excitation power of 400 mW, from the reference InAs QDs in a pure GaAs matrix showing a ground state and two excited states emission peaks. The arrows indicate the calculated transition energies. The electron envelope functions are also shown as insets.

Figure 2 shows the high excitation power 12 K PL data recorded for the reference sample where the QDs are in a pure GaAs matrix. The appearance of multiple peaks results from the states filling effects. The ground state (GS) emission energy is located around 1.086 eV with two excited states at 1.155 eV and 1.224 eV. The appearance of well resolved optical transition energies allows us to estimate the mean size of the buried QDs. Indeed, by tuning the QDs AR, it is possible to fit the calculated ground and excited states transition energies to the experimentally measured PL emission energies.

The calculation has been based on the three emission energies appearing in the PL spectrum. The experimental and calculated emission energies are perfectly matched within some meV yielding an average QD height of 3.8 nm and a base diameter around 28 nm. The calculated wave functions of the first two confined electron states using the estimated realistic size for the buried QDs with an InAs wetting layer thickness of 0.3 nm are shown in the Figure 2. It is important to notice that atomic force microscopy (AFM) investigation of similar uncapped structure revealed a surface density around 2.4 × 10^10^ cm^−2^ and a mean dots’ height of 8 nm with a base diameter around 35 nm giving rise to an aspect ratio (AR) of 0.23. This shows that the values are over estimated. Indeed, the QDs height can be reduced by the capping process [29,30]. Additionally, the AFM technique may also provide an over estimated size due to the tip artifact [31,32]. It is also reasonable to take into account the fact that assuming a pure InAs QDs in the calculation might yields smaller size QDs than the real case where small amount of In atoms may out diffuse.

In order to study the impact the QDs position with respect to the InGaAs SRL, we have conducted a first investigation using these reference QDs parameters. This implies that the InGaAs capping or underlying layer will contribute to the strain reduction without affecting the QDs size. The calculations expect a ground state emission energy redshift around 20 meV upon capping the InAs QDs by 10 nm of InGaAs and around 10 meV when the SRL is inserted below the QDs.

**Figure 3:**
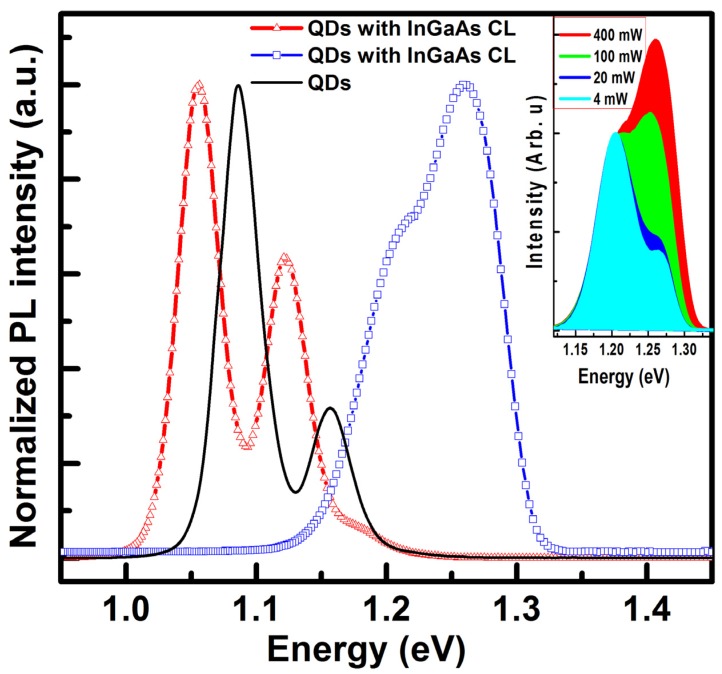
12 K PL spectra taken with an excitation power of 400 mW, from the reference InAs QDs in a pure GaAs matrix and from the InAs QDs with SRL as a cap layer (empty triangles) and as an underlying layer (empty squares). The Inset shows the excitation power dependent PL spectra from the InAs QDs with InGaAs underlying layer. Multiple peaks in each spectra arise from the stat filling effects.

The PL spectra show that capping the QDs by 10 nm (QDCL) leads to a decrease of the PL emission energy by approximately 32 meV as shown by the Figure 3.

The excess energy red shift could arise from the increase of the QDs’ size a result of the strain driven phase separation of the capping layer alloy. This result is in agreement with recently reported structural investigation on similar structures [23]. In the meanwhile, the GS emission energy of the InAs QDs with InGaAs underlying layer (QDUL) shows an unexpected blueshift of the GS emission energy up to 1.204 eV with broadening of the PL linewidth and reduction of the intersublevel spacing energy. A shown by the inset of the Figure 3, the persistence of the excited states emission energy peak at the lower excitation power, testify to a reduced number of coherent QDs [11]. Furthermore, AFM investigation on similar uncapped InAs QDs revealed an increased dots surface density to 4 × 10^10^ cm^−2^, with a broadened size distribution a reduced mean dots’ height down to 5 nm without appearance of bimodal size distribution [11] confirming the later statement. These observations indicate that the mean dots’ size has been seriously altered by the InGaAs underlying layer.

In fact, by varying the dots’ size we succeed in accurately fitting the PL emission energies by the calculated values for both InAs QDs with either SRL capping or underlying layer. The results are summarized in Table 2, where the estimated results using the reference QDs size are shown together with the simulated real dots’ size obtained by tuning the dots’ size to match the experimental transition energies. Accordingly, the calculation reveals an increased buried dots’ size upon capping by InGaAs SRL, while keeping the same aspect ratio. This change of InAs QDs size is explained by the redistribution of Indium and Gallium atoms during the deposition of the InGaAs capping layer on InAs QDs resulted from alloy phase separation phenomena [16].

**Table 2:** Experimental and theoretical Ground state and first two excited states optical transition energies as well as corresponding QD size. The experimental emission energies are given by PL spectroscopy and the average QDs size is estimated through AFM. The expected results refer to the calculated transition energies keeping the simulated buried dot size for the reference sample. The Simulation refers to the realistic dots’ size driven by fitting the theoretical emission energies to the corresponding PL peaks.

Sample	Data	H_QD_/D_QD_	Aspect Ratio (α)	E_0_ (eV)	E_1_ (eV)	E_2_ (eV)
QDs	Experiments	8.5/35	0.24	1.086	1.156	1.224
	Simulation	3.8/28	0.13	1.083	1.151	1.238
QDCL	Experiments	8.5/35	0.24	1.054	1.117	1.194
	Simulation	4/30	0.13	1.051	1.113	1.193
	Expected results	3.8/28	0.13	1.068	1.131	1.213
QDUL	Experiments	5/41	0.12	1.21	1.261	–
	Simulation	2.5/29	0.08	1.204	1.265	1.331
	Expected results	3.8/28	0.13	1.071	1.141	1.217

However, a drastic change of the mean size and aspect ratio is found to occur for the QDs with InGaAs underlying layer. Indeed, the dots’ height is found to decrease to 2.5 nm and the base diameter is increased to 29 nm. In fact, indium segregation during the growth of the SRUL is expected to increase the nominal thickness of InAs material from 2.4 ML to up to 3 ML. Indeed, when the InAs nominal thickness exceeds a critical value, coalesced or/and dislocated QDs are formed and the number and size of active QDs could be critically reduced. This effect is confirmed by detailed PL investigation indicating a degradation of the QDs structural properties [11]. Additionally, the reduced strain increases the InAs critical thickness which is favorable to the formation of thicker wetting layers allowing the growth of smaller size QDs with higher areal density [33].

**Figure 4:**
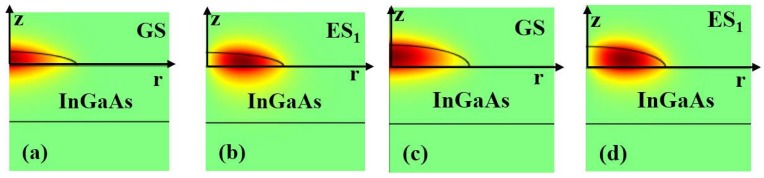
(**a**) and (**b**) indicates the envelop function for electrons with the simulated real size of the buried dots; (**c**) and (**d**) refer to the envelop functions expected for QDs with InGaAs underlying layer (QDUL) with a dot-size that is supposed to be the same as the reference sample.

To further investigate the effect of the size modification that occurred following the growth of the QDs on the top of the InGaAs layer, the calculated electrons wave functions for the ground state and the first excited state are shown by the Figure 4 in the *(r,z) plan*.

Indeed, as a consequence of the decreased realistic size, compared to that of the reference QDs, the electron wave function is found to spread out of the QDs. This behavior indicates less localization as a consequence of the lowering of the effective confining potential barrier and increased confined energy levels.

## 3. Experimental Section

For experimental validation and deeper investigation of the effects of the InGaAs layer’s position on the structural and optical properties of InAs QDs we have used a single InAs QDs layer as reference sample. In the two other samples, the strain has been reduced by an InGaAs layer with 15% indium composition. In the QDCL sample, the QDs were covered by 10 nm InGaAs. However, in the QDUL sample, the InAs QDs layer was deposited on a 10 nm InGaAs. The samples are characterized by PL spectroscopy using a conventional lock-in technique. A 514.5 nm line of an Ar + laser with 0.2 mm spot diameter has been employed as excitation source. More details on the growth process, optical and morphological properties of these samples can be found elsewhere [11].

## 4. Conclusions

In summary, a combination of a simple numerical approach in the frame of the effective mass approximation method and photoluminescence spectroscopy have been employed to perform a detailed investigation of the effect of the InAs QDs position with respect to the strain reducing layer on the buried dots’ size. The numerical calculations using the same dot-size expects a redshift of the emission energies upon underlaying or capping the dots by InGaAs alloy. The PL results show additional redshift for the emission energy of the InGaAs capped QDs and a realistic QDs size simulation revealed an enhancement induced by the alloy phase separation. When the QDs are grown on the top of the InGaAs SRL, the experiments show an unexpected blueshift. The simulation of the buried dots’ size indicated a drastic reduction of the QDs’ height leading to less carrier localization. Our results provide insight towards better understanding and optimization of QDs’ properties by using SRL.
